# Predictive Value of a Combined Model Based on Pre-Treatment and Mid-Treatment MRI-Radiomics for Disease Progression or Death in Locally Advanced Nasopharyngeal Carcinoma

**DOI:** 10.3389/fonc.2021.774455

**Published:** 2021-12-07

**Authors:** Le Kang, Yulin Niu, Rui Huang, Stefan (YUJIE) Lin, Qianlong Tang, Ailin Chen, Yixin Fan, Jinyi Lang, Gang Yin, Peng Zhang

**Affiliations:** ^1^ Department of Radiation Oncology, Sichuan Cancer Hospital & Institute, Sichuan Cancer Center, School of Medicine, University of Electronic Science and Technology of China, Radiation Oncology Key Laboratory of Sichuan Province, Chengdu, China; ^2^ Department of Hematology and Oncology, Anyue County People’s Hospital, Ziyang, China; ^3^ Graduate School, Chengdu Medical College, Chengdu, China; ^4^ Department of Transplantation Surgery, The Affiliated Hospital of Guizhou Medical University, Guiyang, China; ^5^ University of Southern California, Viterbi School of Engineering Applied Data Science, Los Angeles, CA, United States

**Keywords:** radiomics, nasopharyngeal carcinoma, prognosis, prediction model, magnetic resonance imaging

## Abstract

**Purpose:**

A combined model was established based on the MRI-radiomics of pre- and mid-treatment to assess the risk of disease progression or death in locally advanced nasopharyngeal carcinoma.

**Materials and Methods:**

A total of 243 patients were analyzed. We extracted 10,400 radiomics features from the primary nasopharyngeal tumors and largest metastatic lymph nodes on the axial contrast-enhanced T1 weighted and T2 weighted in pre- and mid-treatment MRI, respectively. We used the SMOTE algorithm, center and scale and box-cox, Pearson correlation coefficient, and LASSO regression to construct the pre- and mid-treatment MRI-radiomics prediction model, respectively, and the risk scores named P score and M score were calculated. Finally, univariate and multivariate analyses were used for P score, M score, and clinical data to build the combined model and grouped the patients into two risk levels, namely, high and low.

**Result:**

A combined model of pre- and mid-treatment MRI-radiomics successfully categorized patients into high- and low-risk groups. The log-rank test showed that the high- and low-risk groups had good prognostic performance in PFS (P<0.0001, HR: 19.71, 95% CI: 12.77–30.41), which was better than TNM stage (P=0.004, HR:1.913, 95% CI:1.250–2.926), and also had an excellent predictive effect in LRFS, DMFS, and OS.

**Conclusion:**

Risk grouping of LA-NPC using a combined model of pre- and mid-treatment MRI-radiomics can better predict disease progression or death.

## Introduction

Nasopharyngeal carcinoma (NPC) is epithelial carcinoma originating from the inner layer of the nasopharyngeal mucosa. In 2018, there were 129,000 new cases of NPC in the world (1). The TNM stage system is widely used in risk stratification and therapeutic decision in NPC, and about 70% are diagnosed with locally advanced stage (2). Concurrent chemoradiotherapy with or without induction chemotherapy is the standard treatment with locally advanced nasopharyngeal carcinoma (LA-NPC). However, it is worth noting that there are still significant differences in clinical outcomes among the same TNM stage and similar treatment in LA-NPC; metastasis and recurrence, especially the former, are the considerable causes of treatment failure (3). The 5-year progression-free survival (PFS) for stage III and IVa in NPC were 68.7–87% and 50.4–68%, and the 5-year overall survival (OS) were 75.5–91.4% and 58.3–75%, respectively (4–6). Therefore, developing individualized methods to predict the effect in LA-NPC is necessary.

Radiomics is an algorithm that could automatically extract high-dimensional quantitative features from medical images. These features are extracted from the whole tumor in different ways. They can provide comprehensive information about tumor phenotype, tumor microenvironment, and response to treatment to characterize tumor heterogeneity (7, 8). Magnetic resonance imaging (MRI) was the preferred imaging modality for diagnosis and local stage of NPC (9). Previous studies had shown that MRI-radiomics is an independent risk factor for distant metastasis, local recurrence, and PFS in NPC (10–12). Most of these studies focus on primary tumors of the nasopharynx. A recent study showed that primary tumors and metastatic lymph nodes have different biological characteristics (13). Therefore, it is necessary to consider adding metastatic lymph node information to radiomics based on primary nasopharyngeal tumors.

Due to individualized differences, different NPCs have different responses to chemoradiotherapy, leading to differences in tumor cell populations (i.e., differences in tumor heterogeneity). Currently, there is no literature report on constructing an MRI-radiomics model during chemoradiotherapy to predict LA-NPC. This study aims to screen features associated with PFS labeling in pre- and mid-treatment MRI-radiomics, respectively, to construct a model to predict disease progression or death in LA-NPC (stage III–IVa).

## Material and Method

### Patient

This retrospective study was approved by the institutional review board of our institution. Informed consent from patients was exempted due to the retrospective nature of this study. The experiment included newly diagnosed LA-NPC (stage III-IVa) in Sichuan Cancer Hospital from January 2015 to December 2016. The inclusion criteria were as follows: (1) histologically confirmed LA-NPC (restage according to AJCC 8th edition) and at least one metastatic lymph node. Previous studies associated with head and neck cancer have shown that the radiomics features of increasing the region of interest (ROI) of the lymph nodes provide a better predictive power than those from primary tumors alone (14, 15). According to the definition of Ho et al. (16), the diagnostic criteria of N + include central necrosis, extracapsular spread, the shortest diameter of cervical lymph nodes >10 mm, and the shortest diameter of retropharyngeal lymph nodes >5 mm. (2) pre- and mid-treatment (20 times of radiotherapy) MRI examination of nasopharynx and neck, MRI sequence included axial contrast-enhanced T1 weighted imaging (CET1WI), and axial T2 weighted imaging (T2WI); (3) radical chemoradiotherapy were completed; (4) have available clinical data. The exclusion criteria were (1) motion artifacts, blurring, and in-continuity in MRI images; (2) history of anticancer therapy before baseline MRI scans, such as radiotherapy, chemotherapy, immunotherapy, and surgery; (3) patients with distant metastasis; (4) recurrence or complicated with other malignant tumors; (5) incomplete radiotherapy planning records. Finally, a total of 243 patients were included in further analysis.

Pre-treatment clinical characteristics were collected through the Health Information System (HIS) of Sichuan Cancer Hospital. The characteristics include age, sex, cigarette smoking, alcohol consumption, family history, WHO type, platelet count (PLT), neutrophil count, lymphocyte count, monocyte count, platelet-to-lymphocyte ratio (PLR), neutrophil-to-lymphocyte ratio (NLR), lymphocyte-to-monocyte ratio (LMR), hemoglobin (HB), C-reactive protein (CRP), alanine aminotransferase (ALT), aspartate aminotransferase (AST), lactate dehydrogenase (LDH), alkaline phosphatase (ALP), serum albumin, cumulative dose of radiotherapy, image-guided radiotherapy (IGRT), TNM stage, induction chemotherapy, targeted therapy.

### Treatment

The treatment regimen was concurrent chemoradiotherapy ± induction chemotherapy. The chemotherapy regimen was platinum-based single or dual drug (cisplatin ± paclitaxel), beginning on the first day of radiotherapy. Gross tumor volume (GTV), included both primary nasopharyngeal tumor (GTVnx) and metastatic lymph nodes (GTVln) as demonstrated by clinical, endoscopic, and imaging data. All ROI segmentations were firstly manually performed by a radiation oncologist who had 3 years of experience in NPC radiotherapy and then validated by a senior radiation oncologist who had 10 years of experience. GTV was planned to receive a total dose of 66–76 Gy with conventional fractionation (2.1–2.25 Gy per fraction, five fractions per week). Some patients were treated with anti-EGFR monoclonal antibodies during radiotherapy simultaneously. Nasopharynx and neck MRI were reexamined at 20 times of radiotherapy.

### Follow-Up and Survival Endpoint

MRI scan showed soft tissue swelling or space-occupying and then by histopathology to determine local recurrence. Distant metastasis was diagnosed synthetically by clinical symptoms, physical examination, imaging data, and histopathology. The main endpoint was PFS, while loco-recurrence-free survival (LRFS), distant metastasis-free survival (DMFS), and OS were secondary endpoints. PFS was defined as the time during the tumor progressing (for any aspect) or at death (for any reason) and the first MRI scan. LRFS was defined as the time between the first local recurrence and the first MRI scan. DMFS was defined as the time between the first distant metastasis and the first MRI scan. OS was defined as the time between the death of any cause and the first MRI scan.

### MRI Check

The MRI equipment was Siemens Magnetom Avanto-1.5T/Magnetom Skyra-3T. Some scanning parameters were as follows: T2WI sequence of Magnetom Avanto-1.5T scan was repetition time (TR): 4,890 ms; echo time (TE): 80 ms; field of view (FOV): 340×340 mm; matrix: 320×320 mm; thickness: 3 mm; gap: 3.6 mm. CET1WI sequence was TR: 695 ms; TE: 12 ms; FOV: 300×320; matrix: 320×280; thickness: 3 mm; gap: 3.6 mm. T2WI sequence of Magnetom Skyra-3T scan was TR: 5,290 ms; TE: 85 ms; FOV: 340×340 mm; matrix: 320×320 mm; thickness: 3 mm; gap: 3.6 mm. CET1WI sequence was TR: 769 ms; TE: 12 ms; FOV: 300×320 mm; matrix: 320×280; thickness: 3 mm; gap: 3.6 mm. CET1WI was treated with gadolinium meglumine at a dose of 0.2 mmol/kg.

### Image Acquisition and Segmentation

**Figure 1:**
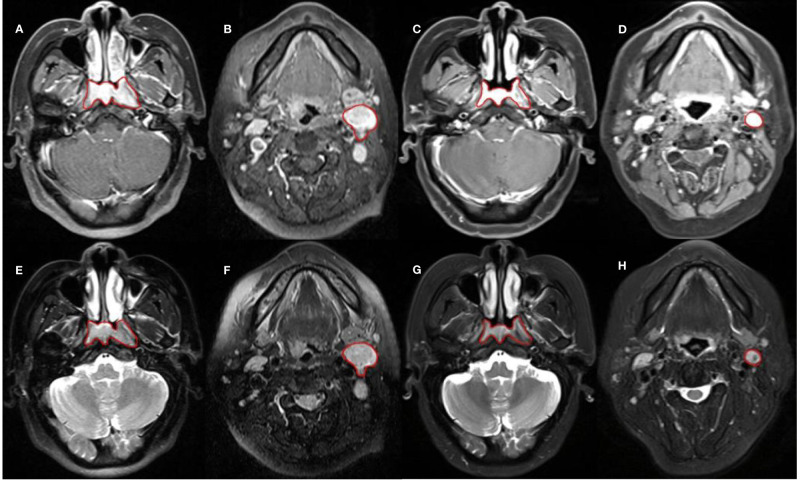
Sketch of ROI. **(A–D)** are CET1WI sequences; from left to right, they are GTVnx and GTVln in pre-treatment, GTVnx and GTVln in mid-treatment. **(E–H)** are T2WI sequences; from left to right, they are GTVnx and GTVln in pre-treatment, GTVnx and GTVln in mid-treatment.

The MRI image was exported through PACS and saved in DICOM format. The saved image was then imported into the MIM planning system for ROI drawing. To ensure the accuracy of the sketch, we used manual segmentation to outline the masses on the CET1WI and T2WI sequence of the primary nasopharyngeal tumor and metastatic lymph nodes in pre- and mid-treatment (as shown in **Figure 1**). The resulting 3D mass area was ROI. In this study, the metastatic lymph nodes with the largest short diameter were selected as the target lesions for GTVln, which is consistent with the study of Bologna (17).

### Image Preprocessing

The uAI Research Portal (Version: 430 sp1) was used to image preprocessing. We processed the image by several filters, including Box Mean, Additive Gaussian Noise, Binomial Blur, Curvature Flow, Box Sigma, Laplacian of Gaussian (LoG), Wavelet, Normalize, Laplacian Sharpening, Discrete Gaussian, Mean, Speckle Noise, Recursive Gaussian, Shot Noise/Poisson Noise filter. In our study, four different LoG filtered images were obtained through different combinations. After three times of wavelet decomposition, the wavelet images of eight various frequency bands were finally obtained, and normalize filter adjusted all MRI images to 255 gray levels in order to standardize the scanning parameters and machinery differences reflected on the images.

### Radiomics Feature Extraction

The uAI Research Portal was also used for feature extraction. Features of different categories were considered: 14 shape features, 18 first-order statistics features, 21 features computed on gray level co-occurrence matrix (GLCM), 16 features computed on gray level run-length matrix (GLRLM), 16 features computed on gray level size zone matrix (GLSZM), 14 features computed on gray level dependence matrix (GLDM), and 5 features computed on gray level dependence matrix (GLDM), a total of 104 radiomics features. The original and filtered image generated 25 groups, so each ROI extracted a total of 2,600 features. Finally, from each image type (CET1WI or T2WI), 2,600 radiomics features were extracted from both the primary tumors and the largest affected lymph node on pre- and mid-treatment, for a total of 20,800 features, namely, 10,400 for pre- and mid-treatment, respectively. Excel S1 and S2 of **Supplementary Materials** shown the all radiomics feature in pre- and mid-treatment.

### Radiomics Feature Selection, Model Building, and Validation

To avoid the influence of class imbalance (85 cases of progress/death *vs* 158 cases of disease-free survival) on the model building, we used a SMOTE algorithm to oversample the original dataset of pre- and mid-treatment, respectively. After amplification, the dataset was randomly divided into a training dataset (476/595) and a test dataset (119/595) according to 4:1. The model building was based on the training dataset, after being preprocessed by center and scale and Box-cox; the feature with no difference between categories was removed. The Pearson correlation coefficient was used to remove redundant features. LASSO regression was used for a further selection of the remaining features, which is consistent with most previous studies (10–12, 18, 19). Then, 20 MRI-radiomics that were most closely related to PFS tags were selected, and the importance of features in the model was sorted. Finally, we selected the top five features respectively to create a radiomics model of pre- and mid-treatment. At the same time, the prediction ability of the model was tested in the training, test, and original dataset by ROC curve and confusion matrix. Eventually, we received the radiomics risk score of pre-treatment named P score and mid-treatment named M score.

### Final Model Development and Risk Stratification

The clinical information, P score, and M score were analyzed by Cox univariate analysis, and we selected the variables with P < 0.05 (bilateral test) to Cox multivariate analysis. According to the results of multiple factors, we chose the variables with P < 0.05 (bilateral test) to train a multivariate Cox proportional hazard regression model, and the predicted values of linear predictive variables of PFS were obtained. The higher the predictive value, the greater the risk of progress/death. The median of the predictive value was used as the threshold for risk stratification. Finally, we compared the Kaplan-Meier survival curves between different risk groups and TNM stages at different clinical endpoints.

### Statistical Analysis

All statistical analyses were conducted using SPSS (version 26.0), GraphPad Prism (version 8), and R software (version 3.5.2). LASSO logistic regression was completed by the “glment” package. The Kaplan–Meier survival analyses were presented by GraphPad Prism. P < 0.05 was considered as statistically significant.

## Result

A total of 243 patients were included for the final analysis. The median follow-up period was 52.7 months (range 10.6–72 months). The specific clinical data were shown in **Table 1**.

**Table 1:** Clinical baseline data of the subjects (N=243).

Clinical features	Percentage/mean ± SD/median (interquartile range)
Age (years)	49.28 ± 10.678
Sex (N/%)	
Male	75.3% (183/243)
Female	24.7% (60/243)
Cigarette smoking (N/%)	
No	53.1% (129/243)
Yes	46.9% (114/243)
Alcohol consumption (N/%)	
No	77.4% (188/243)
Yes	22.6% (55/243)
Family history of cancer (N/%)	
No	93.8% (228/243)
Yes	6.2% (15/243)
WHO histological type (N/%)	
Type I	0.8% (2/243)
Type II	99.2% (241/243)
Neutrophil count (10^9/L)	3.96 (3.08–4.85)
Lymphocyte count (10^9/L)	1.51 (1.22–1.93)
Monocyte count (10^9/L)	0.36 (0.27–0.45)
PLT (10^9/L)	195 (161–244)
HB (g/L)	140 (130–150)
NLR	2.48 (1.95–3.55)
PLR	131.5 (101.4–164.6)
LMR	4.39 (3.25–5.84)
CRP (mg/L)	3.55 (2.28–5.33)
ALT (U/L)	24 (16–34)
AST (U/L)	23 (19–28)
Albumin (g/L)	43.2 (41.4–45.2)
LDH (U/L)	178 (153-205)
ALP (U/L)	82 (69-99)
T stage	
T1	4.1% (10/243)
T2	34.2% (83/243)
T3	29.6% (72/243)
T4	32.1% (78/243)
N stage	
N1	11.1% (27/243)
N2	63.8% (155/243)
N3	25.1% (61/243)
TNM stage	
III	47.7% (116/243)
IVa	52.3% (127/243)
induction chemotherapy	
No	39.1% (95/243)
Yes	60.9% (148/243)
IGRT	
No	16.9% (41/243)
Yes	83.1% (202/243)
Targeted therapy	
No	92.2% (224/243)
Yes	7.8% (19/243)
Cumulative radiation dose (Gy)	44.60 ± 1.11
Clinical endpoints	
None	65.4% (159/243)
Recurrence	14.0% (34/243)
Distant metastasis	18.9% (46/243)
Recurrence and distant metastasis	3.7% (9/243)
Death	21.8% (53/243)

### Establishment and Validation of Pre-Treatment MRI-Radiomics Prediction Model

**Figure 2:**
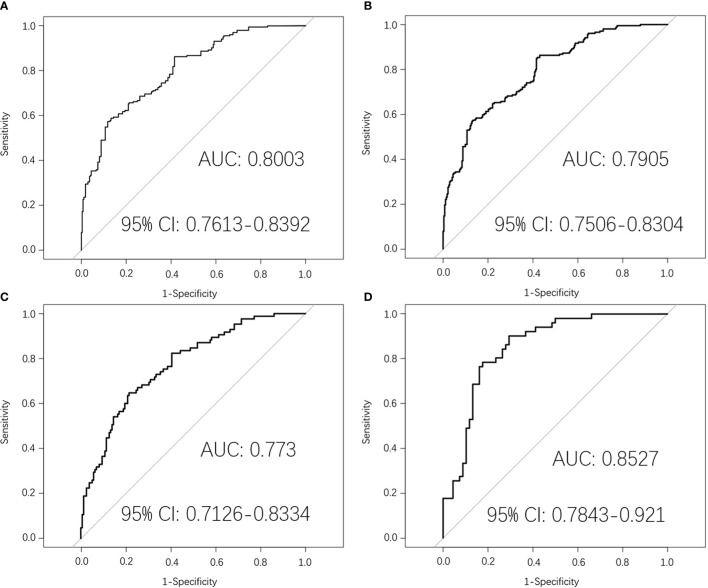
Pre-treatment MRI-radiomics model in each dataset predicted the ROC curves of the PFS in LA-NPC. **(A)** shows the ROC curve of the MRI-radiomics model in the training dataset of pre-treatment, and **(B)** shows the average ROC curve of 10-fold cross-validation of the MRI-radiomics model in the training dataset. **(C, D)** represent the ROC curve of the MRI-radiomics model in the original dataset and test dataset, respectively.

In the pre-treatment prediction model, there were 243 samples in the original dataset, which were expanded to 595 samples by SMOTE algorithm. After randomly grouping according to 4:1, there were 476 samples in the training dataset and 119 samples in the test dataset. Top five of 20 radiomics features were selected, including three from primary nasopharynx tumors and two from metastatic lymph nodes. **Supplementary Figure S1** shown the 20 radiomics feature in pre-treatment. Then the pre-treatment radiomics model to predict PFS in LA-NPC was established by logistic regression. The AUC value of the pre-treatment prediction model in the training dataset was 0.8003 (95% CI:0.7613–0.8392). The average AUC value of five times 10-fold cross-validation in the training dataset was 0.7905 (95% CI: 0.7506–0.8304). The AUC value in the original dataset was 0.773 (95% CI: 0.7126–0.8334). The AUC value in the test dataset was 0.8527 (95% CI: 0.7843–0.921). The ROC curve was shown in **Figure 2**.

**Figure 3:**
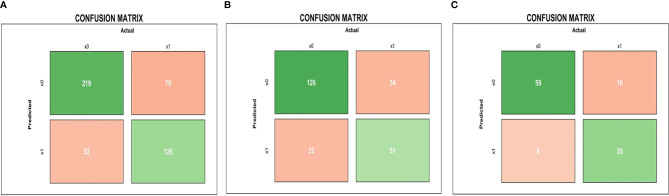
Pre-treatment MRI-radiomics model in each dataset predicted the confusion matrix of PFS in LA-NPC. **(A–C)** represent the confusion matrix of the MRI-radiomics model of pre-treatment in the training dataset, the original dataset, and the test dataset, respectively.

The results of the confusion matrix (**Figure 3**) of the three datasets (training dataset, original dataset, and test dataset) in this study were as follows: the accuracy, precision, sensitivity, specificity, and F1 values of the training dataset were 0.725, 0.704, 0.618, 0.805, and 0.658, respectively. In the original dataset, they were 0.728, 0.614, 0.600, 0.797, and 0.607, respectively. In the test dataset, they were 0.790, 0.795, 0.686, 0.868 0.737, respectively. Finally, according to the weighted coefficient of logistic regression analysis, we obtained a formula for calculating the risk value of each LA-NPC patient:


$$\begin{array}{c} \text{Logit}(\text{P score})=-0.5747+0.7722*\text{normalize}\_\text{firstorder}\_\text{Mean}\_\text{PGTVlnT}1\\ +1.0464*\text{boxmean}\_\text{glcm}\_\text{ClusterShade}\_\text{PGTVnxT}1\\ +0.9113*\text{Log}\_\text{firstorder}\_\text{Log}-\text{sigma}-2-\text{mm}-3\text{D}-\text{Maximum}\_\text{PGTVlnT}1\\ +0.6325*\text{normalize}\_\text{gldm}\_\text{DependenceVariance}\_\text{PGTVnxT}2\\ +0.7434*\text{wavelet}\_\text{glszm}\_\text{wavelet}-\text{HHH}-\text{SmallAreaEmphasis}\_\text{PGTVnxT}2 \end{array}$$

### Establishment and Validation of Mid-Treatment MRI-Radiomics Prediction Model

In the mid-treatment prediction model, the original dataset after oversampling and grouping showed 476 samples in the training dataset and 119 samples in the test dataset. Five radiomics features were selected, including three from primary nasopharyngeal tumor and two from metastatic lymph nodes. **Supplementary Figure S2** shown the 20 radiomics feature in mid-treatment.

**Figure 4:**
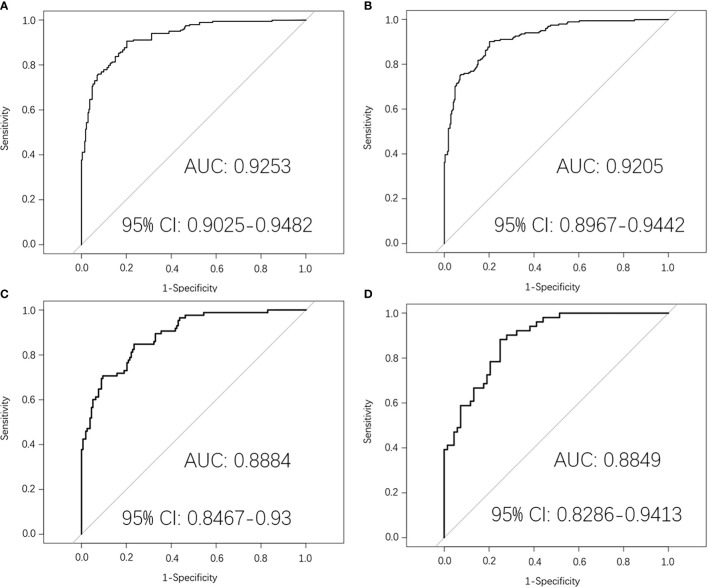
Mid-treatment MRI-radiomics model in each dataset predicted the ROC curves of the PFS in LA-NPC. **(A)** shows the ROC curve of the MRI-radiomics model in the training dataset of mid-treatment, and **(B)** shows the average ROC curve of 10-fold cross-validation of the MRI-radiomics model in the training dataset. **(C, D)** represent the ROC curve of the MRI-radiomics model in the original dataset and test dataset, respectively.

Then the mid-treatment radiomics model to predict PFS in LA-NPC was established by logistic regression. The AUC value of this model in the training dataset was 0.9253 (95% CI: 0.9025–0.9482). The average AUC value of five times 10-fold cross-validation in the training dataset was 0.9205 (95% CI: 0.8967–0.9442), and the AUC value in the original dataset was 0.8884 (95% CI: 0.8467–0.93). The AUC value in the test dataset was 0.8849 (95% CI: 0.8286–0.9413) (**Figure 4**).

**Figure 5:**
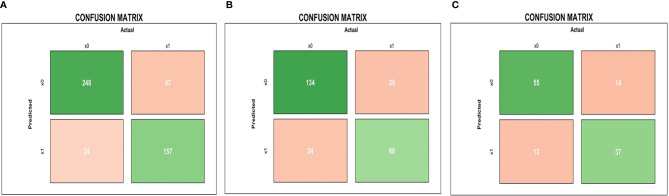
Mid-treatment MRI-radiomics model in each dataset predicted the confusion matrix of PFS in LA-NPC. **(A–C)** represent the confusion matrix of the MRI-radiomics model of mid-treatment in the training dataset, the original dataset, and the test dataset, respectively.

The results of the confusion matrix (**Figure 5**) of the three datasets (training dataset, original dataset, and test dataset) in this study were as follows: the accuracy, precision, sensitivity, specificity and F1 values in the training dataset were 0.851, 0.867, 0.770, 0.912, 0.816, respectively; in the original dataset, they were 0.798, 0.714, 0.706, 0.848, 0.710, respectively; and in the test dataset were 0.773, 0.740, 0.725, 0.809, 0.733, respectively.

Finally, according to the weighted coefficient of logistic regression analysis, we obtained a formula for calculating the risk value of each LA-NPC patient:


$$\begin{array}{c} \text{Logit}\;(\text{M score})=-0.9557-1.5915*\text{shotnoise}\_\text{shape}\_\text{SurfaceVolumeRatio}\_\text{MGTVnxT}1\\ -0.2544*\text{wavelet}\_\text{gldm}\_\text{wavelet}-\text{HLH}-\text{LargeDependenceHighGrayLevelEmphasis}\_\text{MGTVlnT}1\\ -0.5678*\text{wavelet}\_\text{firstorder}\_\text{wavelet}-\text{LHL}-\text{Mean}\_\text{MGTVnxT}1\\ -0.9758*\text{normalize}\_\text{glszm}\_\text{HighGrayLevelZoneEmphasis}\_\text{MGTVlnT}1\\ +1.3875*\text{normalize}\_\text{gldm}\_\text{LargeDependenceLowGrayLevelEmphasis}\_\text{MGTVnxT}1 \end{array}$$

### Final Model Development and Risk Stratification

In univariate Cox analysis, age, alkaline phosphatase, T stage, TNM stage, P score, M score were significantly correlated with PFS. Subsequent multivariate Cox analysis showed that P score (HR: 13.515, 95% CI: 5.185–35.230) and M score (HR: 17.604, 95% CI: 8.113–38.195) were independent risk factors for PFS, as shown in **Table 2**.

**Table 2:** Identification of risk factors of PFS by univariate and multivariate Cox models.

	Univariate Cox regression		Multivariate Cox regression	
	HR (95% CI)	Pvalue	HR (95% CI)	Pvalue
Age (years)	1.028 (1.007–1.050)	0.008	1.011 (0.990–1.033)	0.295
Sex (Female *vs* Male)	1.530 (0.876–2.672)	0.135	–	–
Cigarette smoking (No *vs* Yes)	1.026 (0.671–1.571)	0.905	–	–
Alcohol consumption (No *vs* Yes)	1.282 (0.794–2.069)	0.309	–	–
Family history (No *vs* Yes)	1.747 (0.843–3.621)	0.133	–	–
WHO type (I *vs* II type)	20.421 (0.002–243908)	0.529	–	–
Neutrophil count (10^9/L)	0.957 (0.818–1.119)	0.582	–	–
Lymphocyte count (10^9/L)	1.144 (0.849–1.541)	0.377	–	–
Monocyte count (10^9/L)	0.847 (0.430–1.669)	0.631	–	–
PLT (10^9/L)	1.000 (0.996–1.003)	0.790	–	–
HB (g/L)	1.001 (0.989–1.014)	0.837	–	–
NLR	0.970 (0.856–1.099)	0.632	–	–
PLR	0.999 (0.996–1.002)	0.431	–	–
LMR	1.001 (0.984–1.019)	0.871	–	–
CRP (mg/L)	1.015 (0.988–1.042)	0.277	–	–
ALT (U/L)	0.998 (0.990–1.006)	0.638	–	–
AST (U/L)	0.994 (0.975–1.014)	0.556	–	–
Albumin (g/L)	1.004 (0.999–1.010)	0.134	–	–
LDH (U/L)	1.000 (0.996–1.003)	0.787	–	–
ALP (U/L)	1.004 (1.000–1.008)	0.034	1.005 (0.999–1.011)	0.102
T stage	1.349 (1.056–1.723)	0.016	0.946 (0.687–1.303)	0.734
N stage	1.411 (0.978–2.035)	0.066	–	–
TNM stage (III *vs* IVa)	1.915 (1.223–3.000)	0.005	0.697 (0.397–1.225)	0.210
IC (No *vs* Yes)	0.861 (0.554–1.340)	0.507	–	–
IGRT (No *vs* Yes)	1.035 (0.584–1.837)	0.905	–	–
Targeted therapy (No *vs* Yes)	1.244 (0.574–2.696)	0.580	–	–
Cumulative radiation dose (Gy)	0.966 (0.792–1.179)	0.737	–	–
P score	24.257 (10.375–56.716)	0.000	13.515 (5.185–35.230)	0.000
M score	23.046 (11.678–45.478)	0.000	17.604 (8.113–38.195)	0.000

**Figure 6:**
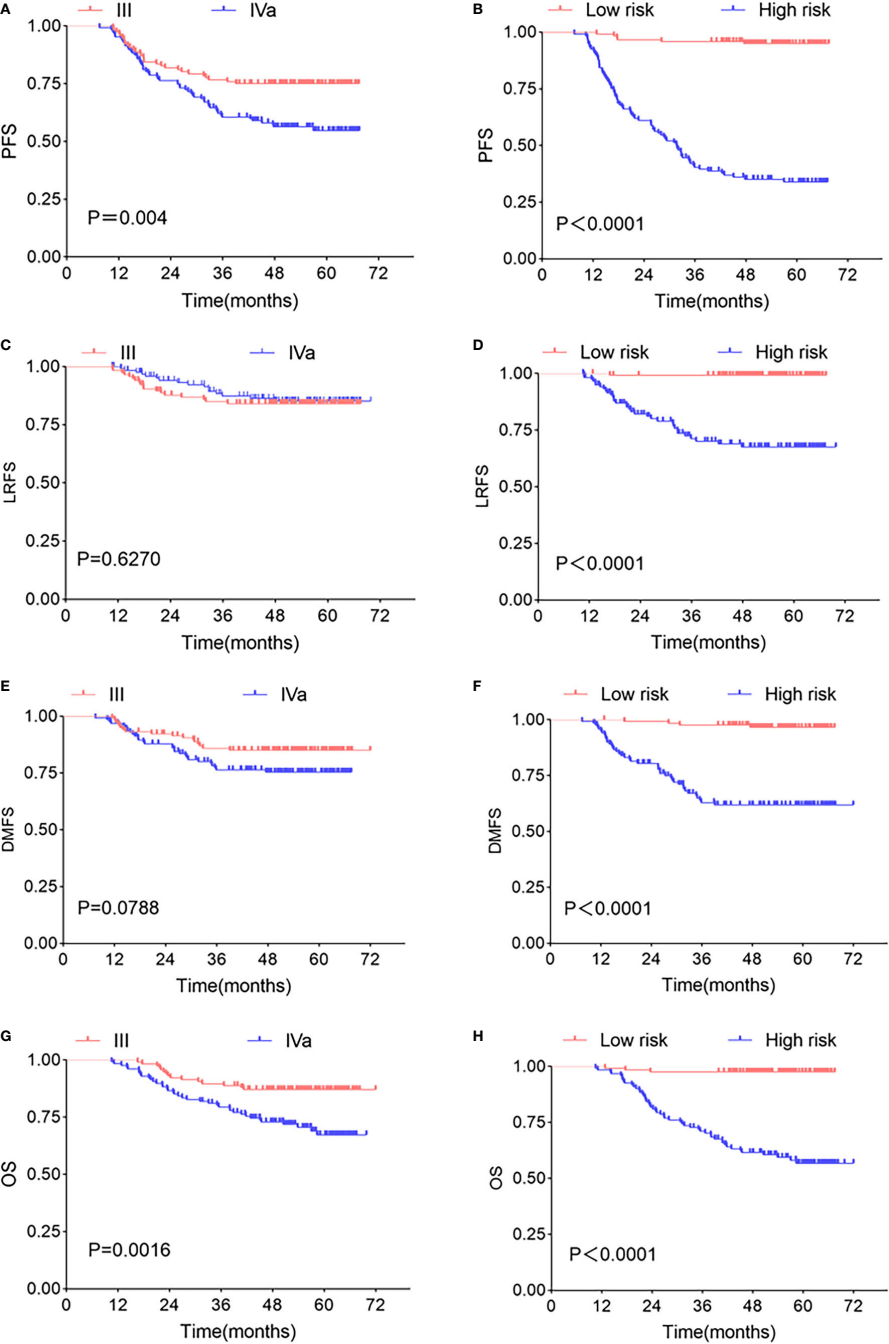
Kaplan-Meier survival curve. Kaplan-Meier survival curves of TNM stages and two risk groups at different clinical endpoints. TNM stages in PFS **(A)**, LRFS **(C)**, DMFS **(E)**, OS **(G)** survival curve, high- and low-risk groups in PFS **(B)**, LRFS **(D)**, DMFS **(F)**, OS **(H)** survival curve. The P-value in the figure is obtained by the log-rank test.

We put P score and M score into multivariate Cox regression model, and the predicted values of PFS linear predictive variables were obtained. The median predicted value was used as a threshold to classify high- and low-risk patients. In terms of prognostic power for PFS, the high- and low-risk groups (P<0.0001, HR: 19.17, 95% CI: 12.77–30.41) was significantly prognostic than TNM stage (P=0.004, HR: 1.913, 95% CI: 1.250–2.926). Similar results could be found by looking at the Kaplan-Meier curves for LRFS, DMFS, OS of the high-/low-risk groups and TNM stage, for as far as LRFS is concerned, the log-rank test showed P < 0.0001 (HR: 44.61, 95% CI: 22.60–88.05), P=0.6270 (HR: 0.8464, 95% CI: 0.4321–1.658), respectively; for DMFS concerned, P < 0.0001 (HR: 14.11, 95% CI: 7.864–25.30) and P=0.0788 (HR: 1.700, 95% CI: 0.9536–3.030), respectively; for OS concerned, P < 0.0001 (HR: 20.18, 95% CI: 11.75–34.66), P=0.0016 (HR: 2.532, 95% CI: 1.478–4.339), respectively (**Figure 6**).

## Discussion

In recent years, radiomics has developed rapidly in medicine, and good results have been achieved in predicting the effect of tumors. MRI is a standard imaging method in NPC, and it has unique advantages. First of all, MRI can provide superior anatomical information (such as spatial location) and has good soft tissue contrast-detection ability. Secondly, different MRI sequences may be sensitive to critical components of tumor physiology, such as blood flow and cell density, and MRI also can distinguish regions in the tumor that contain different environments that may affect local cell phenotypes and genotypes, such as blood flow changes. Finally, MRI can be the non-invasive and repeated examination of the tumor to evaluate the treatment response to be integrated into the treatment strategy. So, the MRI image was used to establish the LA-NPC prediction model through radiomics. This study explored the value of MRI-radiomics features on pre- and mid-treatment in predicting effect in LA-NPC. The results showed that the M score and P score were independent prognostic indexes of PFS. Finally, we put them into the multivariate Cox model to calculate the risk score. We successfully stratified the risk of the LA-NPC. Through the Log-rank test, we found that MRI-radiomics showed good predictive ability in PFS, LRFS, DMFS, and OS.

By screening the pre-treatment MRI-radiomics features, we got 20 radiomics features related to PFS in LA-NPC. It is better to consider that the ratio between the amount of data and the number of features that can be accommodated by logistic regression is more than 20:1 (20). We selected the top five features to establish a pre-treatment prediction model, and the risk score named P score was calculated (21). In previous studies, an MRI-based model on primary nasopharyngeal tumors had been proved to be a significant prognostic biomarker for PFS in LA-NPC (22, 23). Furthermore, the research by Yang et al. indicated that an MRI-based model on metastatic lymph nodes is a significant risk factor for PFS in LA-NPC (24). Thus, MRI-radiomics features from both metastatic lymph nodes and primary nasopharynx tumors contribute to PFS prediction in LA-NPC, which is consistent with our research. As far as we know, there is no related research on the radiomics features of mid-treatment. Similarly, we calculated the risk score of mid-treatment named M score. The MRI-radiomics model of pre- and mid-treatment was internally validated by 10-fold cross-validation in the training dataset. The average AUC values were 0.7905 (95% CI: 0.7506–0.8304) and 0.9205 (95% CI: 0.8967–0.9442), respectively, which indicates that the model has good repeatability. In addition, the two models have high AUC values in both original and test datasets (**Figures 2**, **4**), which shows that the model has good generalization ability and portability. Furthermore, the performance of the two models in the confusion matrix in different datasets (**Figures 3**, **5**) is also outstanding.

Comparing the radiomics features included in the two models, the pre-treatment prediction model had two first-order features (average eigenvalues and maximum eigenvalues) and three texture features (GLCM, GLDM, GLSZM); the mid-treatment prediction model had one shape feature (surface area/volume ratio), one first-order feature (average eigenvalue), and three texture features (GLDM, GLSZM). The shape features reflect the volume, sphere, surface area/volume ratio of the tumor. Previous studies had found that primary tumor volume is closely related to local control, distant metastasis, and OS in NPC (25). Zhang et al. worked on the development and validation of an MRI-based model (including surface area/volume ratio) for predicting distant metastasis of NPC. The model has good evaluation ability in the validation cohort (C index: 0.74, 95% CI: 0.58–0.85) (11). First-order statistical features are the simplest statistical descriptors, including gray average, maximum, minimum, variance, percentile, etc. (24). GLCM can reveal the spatial complexity of tumors and may provide information about central necrosis or tumor metastasis-dependent factors, such as yes-related proteins (13). Several studies had shown that GLCM is closely related to the recurrence, metastasis, and OS of NPC (10–12, 17, 18, 24, 26). Zhang et al. demonstrated that GLSZM is associated with the risk of distant metastasis of NPC (10). Farhan et al. found significant differences between recurrent and non-recurrent regions in seven features (including GLSZM) in the radiomics analysis of intratumoral spatial heterogeneity in LA-NPC (19). GLDM quantifies the dependence between the gray values of adjacent pixels and the gray values of central pixels within a certain distance, and its predictive value in NPC had been confirmed by Zhang et al. (10).

We also found that three of the features in the pre-treatment prediction model came from CET1WI, and two were from T2WI, while all the features of the mid-treatment prediction model came from CET1WI. By comparing the accuracy, precision, sensitivity, specificity, F1 value, and AUC value of the two models, we noticed that the mid-treatment prediction model is better than the pre-treatment in training and original dataset, which may indicate that T2WI mainly reflects the density and boundary of the tumor. However, CET1WI reflects the heterogeneity and structure within the tumor (such as tumor angiogenesis) (27), which is crucial for judging the prognosis. Zhang et al. also found that the contribution of CET1WI to the model is more significant than that of T2WI (11), which is consistent with the results of their another study (the radiomics prediction based on CET1WI sequence is better than T2WI sequence or combined with CET1WI and T2W sequence) (28). Jiang et al. also proposed that using CET1WI to build a model produces better results than T2WI (29).

The features’ inconsistency between pre- and mid-treatment prediction model is attributed to LASSO regression. In the screening radiomics features, LASSO regression will compress some relatively unimportant features, adjust the coefficients to zero for insignificant parameters, and rank the importance of features, for example, “wavelet_firstorder_wavelet_LHH-Mean_GTVnxT1” ranks thirteenth in the Pre-treatment prediction model and sixteenth in the mid-treatment, showing the features included in the pre-treatment model are not entirely useless, just their importance has changed. It also indicates that the tumor cell population has changed after chemoradiotherapy, leading to changes in heterogeneity within the tumor.

We compared the Kaplan-Meier survival curves between different risk groups and TNM stages at different clinical endpoints. The results showed that the high- and low-risk group had an excellent ability to predict PFS (P<0.0001 HR: 19.17, 95% CI: 12.77–30.41) was better than the TNM stage (P=0.004, HR: 1.913, 95% CI: 1.250–2.926). The MRI-radiomics model’s ability to predict the LA-NPC effect is better than the TNM stage had been confirmed in some studies, consistent with our study (12, 18, 26). Interestingly, we tested the high- and low-risk group at other endpoints and found that they all performed well in LRFS, DMFS, and OS, which was similar to some of the results of Marco Bologna (26), who used OS as the label for radiomics features screening, and the final prediction model also had good predictive ability in LRFS. In the study, our radiomics features were labeled with PFS, which includes patients with recurrence, metastasis, and death according to the definition, so the features we screened have predictive values for different endpoints.

Marius suggested several considerations when conducting radiomics studies (30). Firstly, in addition to randomized clinical trials, the class imbalance is common, especially in retrospective studies using routine clinical data. There is little uniformity between interesting and non-interesting events in the cohort. For example, in our study, about 35% of patients had events of interest (progress/death). When evaluating MRI-radiomics features to predict PFS in NPC, we must take the imbalance between the percentage of patients with and without interesting events (35%) into account. The classifier that assigns all the cases in the sample to the “no event of interest” group seems to have a 65% correct rate. Still, it doesn’t make clinical sense because it cannot actually distinguish whether interesting events have occurred by MRI in LA-NPC. Therefore, the overall accuracy and sensitivity, specificity, AUC value should be reported. Our study also used a SMOTE algorithm to balance the impact of class to reduce data imbalance on the research (31). Secondly, overfitting occurs when a model with many input parameters or too many degrees of freedom “memorizes” data. In addition to the features related to disease, the model also contains features reflecting image noise and random fluctuations. Generally, there are two processing methods: reducing the number of features, or performing regularization on the data. Here we compared the Pearson correlation coefficients to check and avoid collinearity between variables, and used LASSO regression for feature selection to avoid overfitting. Besides, the SMOTE algorithm balances the class distribution by synthesizing a small number of samples, which reduces the possibility of overfitting.

This study has two main advantages. Firstly, our research is the only one that demonstrates the predictive effect of the mid-treatment radiomics features on PFS in LA-NPC. We found that the use of radiomics information of mid-treatment can more comprehensively evaluate the response of LA-NPC to treatment and better evaluate the prognosis. On the other hand, we indirectly confirmed that the heterogeneity of tumors would change during chemoradiotherapy. The Cox model combined the pre- and mid-treatment radiomics features for risk stratification and found an excellent predictive effect across different clinical endpoints. Secondly, it had been proved that the population of different genomes is one reason for the clinical heterogeneity of radiotherapy efficacy (32). It is well known that radiomics is assumed to represent the histological heterogeneity of solid tumors (33). Although more than 90% of LA-NPC had positive lymph nodes, previous studies ignored metastatic lymph nodes (22, 23). We also collected the radiomics features of primary nasopharyngeal tumors and metastatic lymph nodes to describe tumor biological characteristics better.

This study also has some limitations. Firstly, this study is a retrospective study conducted by a single agency in non-endemic areas of NPC and lacks external validation. It is necessary to perform a large-sample multicenter prospective validation in NPC endemic and non-endemic regions to obtain strong evidence of clinical application. Secondly, the disunity of the treatment plan will also affect the prediction effect of the model. Finally, MRI-radiomics models and statistical analysis algorithms are unfamiliar and complex to the clinic. To solve this problem, we can set up a website or application, and doctors can upload images and clinical variables to obtain results.

## Conclusion

The MRI-radiomics model (pre- and mid-treatment) is a powerful tool to predict the disease progression/death in LA-NPC. We calculate the risk score of disease progression/death in LA-NPC by combining the radiomics characteristics of pre- and mid-treatment and stratify the patients with high and low risk, which can not only predict the PFS in LA-NPC but also predict the LRFS, DMFS, and OS.

## Data Availability Statement

The original contributions presented in the study are included in the article/**Supplementary Material**. Further inquiries can be directed to the corresponding authors.

## Author Contributions

PZ, GY, LK, and YN designed this study. LK, YN, RH, SL, QT,AC, and YF conducted the study and analyzed the results,development of the model, and drafted the manuscript under the supervision of JL, PZ, and GY. LK took part in the drawing target outline, data extraction, development of the model, YN took part in the research general design, data extraction, development of the model,and they carried out the main part of the study, they contributed equally to this work and share first authorship. PZ and GY have contributed equally to this work and share corresponding authorship. The remaining authors are ranked by their contribution to research. All authors contributed to the article and approved the submitted version.

## Funding

Key research and development Program of Science and Technology Department of Sichuan Province “Application of Multimodal Radiomics and Artificial Intelligence in Precise Treatment of Head and neck Tumors”, item no. 2019YFG0185. Liangshan science and Technology Bureau of Sichuan provence, item no.18YYJS0094. Key R & D support Plan of Chengdu Science and Technology Bureau,item no.2021-YF05-02382-SN.

## Conflict of Interest

The authors declare that the research was conducted in the absence of any commercial or financial relationships that could be construed as a potential conflict of interest.

## Publisher’s Note

All claims expressed in this article are solely those of the authors and do not necessarily represent those of their affiliated organizations, or those of the publisher, the editors and the reviewers. Any product that may be evaluated in this article, or claim that may be made by its manufacturer, is not guaranteed or endorsed by the publisher.
