# Supplementary material for: Predictive Value of a Combined Model Based on Pre-Treatment and Mid-Treatment MRI-Radiomics for Disease Progression or Death in Locally Advanced Nasopharyngeal Carcinoma
Source: Front Oncol. 2021 Dec 7;11:774455. doi: 10.3389/fonc.2021.774455 (PMC8688844; doi:10.3389/fonc.2021.774455)

Supplementary Material

# Supplementary Data

excel S1 and S2 of Supplementary Materials shows the all radiomics feature in pre-and-mid-tretment

# Supplementary Figures and Tables

## Supplementary Figures.

Supplementary Fig. S1 shows the twenty radiomics feature in pre-tretment


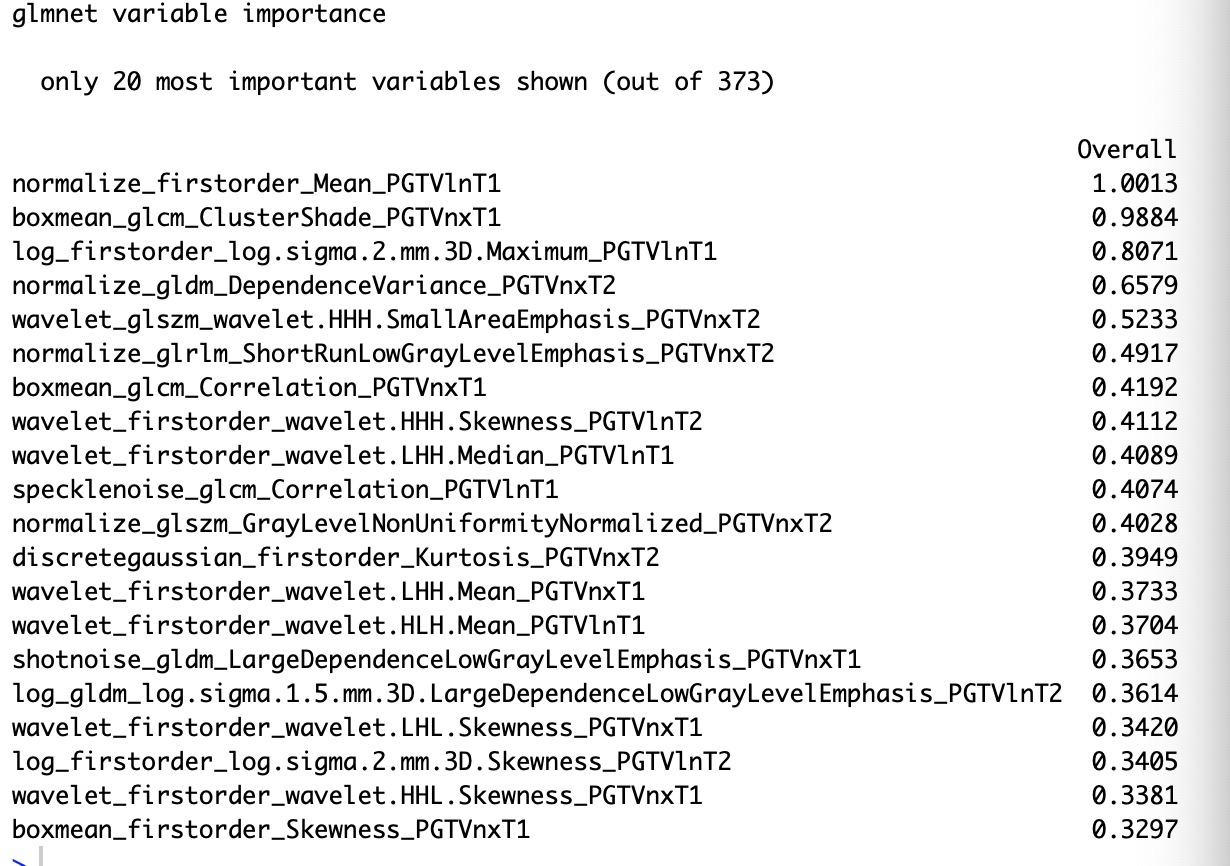


Supplementary Fig. S2 shows the twenty radiomics feature in pre-tretment


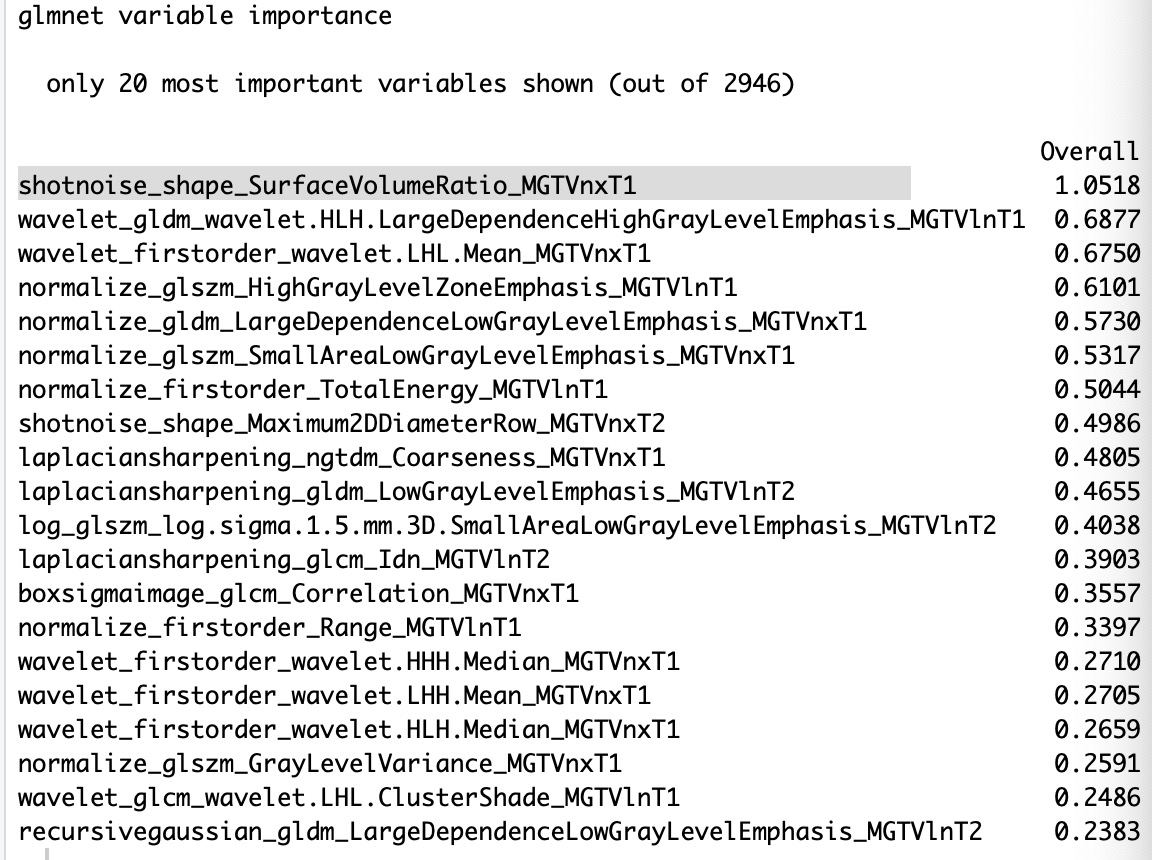

Supplement: Supplementary file 1 [file DataSheet_1.zip › Supplementary_Material.docx]
